# A tool for the assessment of the risk drivers and public perception of WASH in South Africa

**DOI:** 10.4102/jamba.v16i2.1782

**Published:** 2024-10-31

**Authors:** Phindile Madikizela, Janice Limson, Ronen Fogel, Jozef Ristvej, Roman Tandlich

**Affiliations:** 1 Disaster Management and Ethics Research Group, Faculty of Pharmacy, Rhodes University, Makhanda, South Africa; 2 Biotechnology Innovation Centre, Faculty of Science, Rhodes University, Makhanda, South Africa; 3 Department of Crisis Management, Faculty of Security Engineering, University of Žilina, Žilina, Slovakia

**Keywords:** WASH, online search volumes, plugin, disaster risk, rapid assessment, public interest

## Abstract

Temporal trend analysis of the Google-search volumes and terms related to water, sanitation and hygiene (WASH) in South Africa was performed using a computer plugin between January 2004 and June 2022. This study was conducted as WASH has played an important role in the containment of the recent coronavirus disease 2019 (COVID-19) pandemic, and it is also one of the most effective and easiest-to-deploy tools in decreasing risk from infectious diseases. For the WASH-related terms, the monthly search volumes ranged from the minimum average of 480 for pit latrines to the maximum of 30236 for diarrhea or diarrhoea for the studied period. The Spearman correlation coefficients ranged from –0.29462 to 0.96647, with the *p*-values ranging from 0.00001 to 0.28789. On a yearly basis, there was a direct correlation between the WASH-related search volumes extracted and the access of the South African population to basic water and sanitation. There was an inverse relationship between the WASH-related search volumes extracted on an annual basis and the death rates from diarrhoeal diseases among children under 5 years of age in South Africa between 2004 and 2020. Results of the current study indicate that a Google-derived search volume can be useful in the assessment of the public’s interest in WASH-related topics in South Africa.

**Contribution:**

Therefore, the study findings could be used to optimise the design and targeting of public awareness campaigns on WASH during the coronavirus pandemic or similar infectious disease burdens and related disaster risks.

## Introduction

The recent coronavirus disease 2019 (COVID-19) pandemic showed that personal hygiene measures are some of the most effective and easiest-to-deploy tools in decreasing the risk of infectious diseases. Tandlich et al. (2024) analysed the trends in disaster risk and vulnerability of the South African population in terms of water, sanitation and hygiene (WASH) from 1990 until 2015. The South African national strategies to improve the WASH situation of the population have been implemented and continue to be implemented in line with the government’s commitments (Masindi & Dunker 2016; Tandlich et al. 2024). The significance of such efforts is further amplified in the context of the sustainable development goals (SDGs), as there were still shortcomings in the delivery of SDG 6 – related to WASH (South African Government 2019). Some shortcomings are still prevalent, for example only 52%–58% of water sources complied with the water quality guidelines and regulatory requirements (South African Government 2019:71–74). Masindi and Dunker (2016) found that up to 35% of potable water in South Africa was not paid for in 2014 (Masindi & Dunker 2016). These findings point to infrastructure and service delivery problems in South Africa in relation to WASH and related health risks to the South African population. Causes for the shortcomings can include insufficient maintenance of water and sewage treatment plants and the increasing backlog in recent years (Masindi & Dunker 2016). The results of Tandlich et al. (2024) indicated that there was little to no significant correlation between the disaster risk from waterborne diseases and WASH-related vulnerability in South Africa during 1995 and 2015. Obstacles to the implementation of the WASH in Southern Africa include the lack of awareness and community involvement (Tseole et al. 2022:e0271726).

Understanding the levels and drivers of the public’s interest in WASH is critical to the success of any strategies to address the shortcomings, raise awareness about WASH and mitigate the impacts. The current study is aimed at investigating the temporal trends in the South African population’s interest in WASH using Internet search engine data from Google for the January 2004–June 2022 period. The working hypothesis of the current article is that the disaster risk from waterborne disease and the direct impact of the WASH circumstances on the existence of the South African population will influence the interest of the South African population in WASH-related topics. The sequence of the methodological steps in the article starts with the extraction of the search volumes from Google for the WASH-related terms search trends in South Africa from January 2004 until the end of June 2022. Then, the extracted data are subjected to statistical testing and correlation analyses with parameters that characterise disaster risk from waterborne diseases, access to improved water and sanitation and hygiene facilities. Finally, the representativeness of the information derived from the WASH-related Google search terms is analysed, and wider implications of the study results are suggested.

## Research methods and design

To accomplish the study’s objectives, a detailed account of all the study’s methodological steps is provided below. These steps will cover the Google-related WASH data extraction, analysis of such data and conclusion formulation.

### Search trends in the WASH-related terms in South Africa and their links to disaster risk

The search volumes for monthly interest in WASH-related topics by the South African population were performed using the Keywordseverywhere.com plugin and an approach similar to Tandlich et al. (2023:21–26). The plugin was recently partially validated for providing reliable data by Madikizela (2023:Chapter 1). The Google searches, and extraction of the monthly search volumes on Google (Burivalová, Butler & Wilcove 2018:509–514; Tandlich et al. 2023:21–26), were performed for the following keywords and search terms/phrases: sanitation, WASH, diarrhoea or diarrhea, hygiene, water and drinking water, sewage, greywater/grey water, pit latrine and toilet. The monthly search volumes were extracted from the beginning of 2004 until the end of June 2022. The monthly search volumes will be based on a sliding average of Google searches in a given country and month. In other words, the monthly volumes will be recalculated on an ongoing basis and might change as time progresses (Burivalová et al. 2018:509–514; Pretorius, Kruger & Bezuidenhout 2022:53–69). The potential change in the absolute monthly values of search volume was estimated by data extraction on two separate occasions. The coefficient of variation (COV) in the values of the extracted search volumes was calculated using Equation (1):


$$COV=100\times \frac{\left|Value-Average\right|}{Average}$$ [Eqn 1]

where:

*COV* = the percentage of change in the search volumes that were extracted for the particular search term on two separate occasions, but for the same time period from January 2004 until June 2022.

*Value* = represents the particular monthly value of the term that was extracted on a given occasion.

*Average* = the average term between monthly volumes that were extracted on two occasions for the same time period from January 2004 until June 2022.

*The numerator* = the absolute value of the difference between the average of the two extracted values for the same month in a given calendar year.

After the *COV* calculations, the monthly search volumes were analysed for any statistically significant correlation with the search history or time (month number 1 was January 2004 and month number 222 was June 2022). The analysis was done using the Spearman coefficient at a 5% level of significance (https://www.socscistatistics.com/tests/spearman/default2.aspx). Search history was the independent variable representing time, and the search volume was the dependent variable. If the monthly search volumes for a particular search term were increasing or decreasing with time, then the Spearman correlation coefficient would be directly or inversely proportional to the search history or time, and any such correlation would be statistically significant. To characterise the distribution of the values of the individual search volumes, the following numbers were calculated using Microsoft Excel 2016 (Johannesburg, South Africa): the arithmetic average, the median and the mode. Any potential differences in the South African public’s interest in the individual nine search terms, which were used to characterise the WASH, were analysed using the Kruskal-Wallis analysis of variance by ranks at a 5% level of significance (Hammer, Harper & Ryan 2001:1–9).

As WASH impacts the health of the South African population, it is reasonable to expect that interest in WASH-related topics would be in part driven by or related to the human health impacts of WASH in South Africa. This can be expressed through proxy indicators of the WASH-related disaster risk, namely using the mortality of children under 5 years of age (*MCUFRY*; Tandlich et al. 2024). Data on the access of the South African population to improved/water and sanitation facilities, as well as the *MCUFRY*, were extracted from the World Bank (2022a, 2022b, 2022c). In addition, the South African population’s access to hand-washing facilities was extracted from the General Household Survey of Statistics South Africa and the World Bank database (Table 3 and Appendix 1 - Table 1-A1). The annual access to these resources was correlated with the yearly search volumes for the sanitation terms. For all nine search terms for WASH, the monthly volumes were summed up across the given calendar year, and then all yearly search volumes were added up to obtain the total number of the WASH-related term searches in South Africa, as shown in Equation (2):


$$YTWRMT=\Sigma_{1}^{9}\Sigma_{1}^{12}\;X_{i}$$ [Eqn 2]

where:

*YTWRMT* = the total search volume of the WASH-related terms in a given calendar year by the South African population, that is values were obtained for 2004, 2005, 2006,…, 2020.

$\Sigma_{1}^{9}$ = indicates the summation of all nine WASH-related terms that were searched for and that the monthly search volumes were extracted for a given calendar year.

$\Sigma_{1}^{12}$ = indicates the summation of the search volumes of the South African population’s interest for the particular one of nine WASH-related search terms over the 12 months of the given calendar year.

*X*_*i*_ = stands for the monthly search volume in South Africa for sanitation, WASH, diarrhoea or diarrhea, hygiene, water and drinking water, sewage, greywater or grey water, pit latrine or toilet.

Hygiene was considered a more encompassing term as hand washing/hand sanitiser, and it was included here. At the same time, it is not important in the authors’ opinion to differentiate between the public’s interest in the wash standards or WASH as a more general term, as they will reflect similar meanings in the South African public’s interest in searching for the WASH-related topics on Google. The *YTWRMT* values were then correlated using the access to water, sanitation and hygiene facilities through the Spearman correlation coefficient. Based on data availability, the correlations were only performed for the 2004–2020 time period. The qualitative drivers of the interest of the South African public in the WASH-related terms using the related keywords, the ‘people also searched for’ and the long-tail keywords from the Keywordseverywhere.com plugin. To examine the development of the qualitative drivers over time, the keywords were examined over 12 months (October 2021–October 2022), 5 years (October 2017–October 2022) and the 2004–2022 period.

### Relative interest in WASH significance in South Africa compared to other search terms

The Google-derived data from the plugin of Keywordseverywhere.com can be looked at as a form of a public interest sample of the South African population’s opinions on WASH-related in a particular calendar month. The authors wanted to know what percentage of the total number of searches on Google in South Africa were represented by the searches in the WASH-related terms. This could provide an indication of the level of priority that the South African public assigns to WASH as a topic in comparison to other topics. To do this, the *YTWRMT* values were expressed as a percentage of all Google searches in South Africa in a given year. That term was designated as *PTSSA* in the further text of the current article, as defined in Equation (3):


$$\begin{array}{l} PTSSA=100\times \frac{YTWRMT}{TGSGY\times \frac{TPSA}{TWP}\times \frac{PSAPIA}{PWPIA}}=100\times \\ \;\frac{YTWRMT\times TWP\times PWPIA}{TGSGY\times TPSA\times PSAPIA} \end{array}$$ [Eqn 3]

where:

*YTWRMT* = the total search volume of the WASH-related terms in a given calendar year by the South African population, that is values were obtained for 2004, 2005, 2006,…, 2020.

*TGSGY* = the total number of all Google searches performed in a given calendar year worldwide.

*TPSA* = the total South African population in a given calendar year.

*TWP* = the total global population in a given calendar year.

*PSAPIA* = the percentage of the South African population who had access to the Internet or who were using the Internet in a given calendar year in Equations (2) and (3).

*PWPIA* = the percentage of the world population, which used the Internet in a given calendar year.

The total population in a given calendar year for the study period, as well as the total population for South Africa, were extracted from the World Bank data in graph format or from the CSV format (data for South Africa; World Bank Data 2022e). In an analogical fashion, the data for the percentage of the population using the Internet was extracted from the World Bank Database from 2004 until 2020 (World Bank Data 2022f). The total number of Google searches in a given year was extracted from literature for the 2004–2016 period (Burivalová et al. 2018:509–514). For the 2019–2022 period, the total yearly search volumes for all of Google and the global scale were estimated by multiplying daily estimates of the total Google searches by 365/366 in a particular calendar year (Internet Live Stats 2022; Skai 2019). For 2019 and 2022, the number of total Google searches was equal to 2 trillion. At the same time, the number of core searches on Google has levelled off since about 2016 (Statista 2022). The last source is a newspaper article or online source, which has not been subjected to academic scrutiny/peer review. However, the use of these data is the only way to obtain real-world estimations of the total search volumes on Google in line with previous reports (Burivalová et al. 2018:509–514). Therefore the values of the total number of Google searches in a given year were assumed to be constant between 2016 and 2022. As a result, the total yearly volume of searches for the 2017–2020 period was assumed to be equal to the average number for the 2016–2022 period. At the same time, the calculations for Equation (3) were conducted for the 2004–2020 period based on data availability. *PTSSA* can provide an indication to disaster risk management practitioners and public health officials about the relative interest of the public in WASH and how that interest has developed or fluctuated over time. By comparing these parameter values with the levels of service delivery, potential planning solutions to tackle outstanding challenges in the WASH challenges can be devised in South Africa.

### Sample size estimation of the Google search terms as a tool to assess public opinion

In addition to the calculated *PTSSA* values, the WASH-related search volumes on Google by the South African public were compared to the necessary sample size from a survey on the WASH-related topics in the country. The necessary sample size was calculated for a 95% confidence level, 5% margin of error and 50% proportion (see https://www.calculator.net/sample-size-calculator.html for details; website accessed on 24th August 2024). The estimation of the sample size and its comparison with the public interest in search volumes could assist disaster risk management practitioners and public health officials in assessing the likely value of the Keywordseverywhere. com plugin as a tool to gauge public opinion in South Africa. This is important as the surveys of public opinion require time to be organised and the results take some, even though potentially minimum amount of, time to be evaluated. After that the conclusions must be drawn, and policy or assistance action taken. The Google search terms are available, and the necessary analysis can be done much faster. Therefore if each search volume is assumed to be the result of one click and that one search or click is assumed to be performed by one person, then the Keywordseverywhere.com plugin could be used as a real-time surrogate for the need to run a survey on topics. This tool will, however, only provide a high-level idea and not a specific targeted picture that specific survey questions might.

## Results and discussion

Analysis of the monthly search volume from Google was conducted using the Keywordseveerywhere.com plugin. Data extraction was performed on two occasions from South Africa for the time period from January 2004 until the end of June 2022. The extracted data were subjected to statistical testing, and the findings are used to provide a broader context for the study implications.

### Analysis of the trends in the search trends in WASH-related terms in South Africa

The search volumes for the WASH-related terms were extracted on two occasions between June 2022 and August 2022, with results shown in Table 1. It can be seen from the data that the monthly search volumes ranged from the minimum average of 480 for pit latrines to a maximum of 30 236 for diarrhea or diarrhoea. The average *COV* value ranged from 1.3 ± 1.3% for toilets to a maximum of 22 ± 6% for sanitation. Based on the Spearman correlation coefficient results, there was a statistically significant increase in the South African public’s interest in the following WASH-related terms with the search history or time between 2004 and June 2022: sanitation, WASH, diarrhoea or diarrhea, water or drinking water, pit latrine and toilet. There was no trend in the monthly search volumes with time for greywater or grey water and decreasing interest was indicated for the South Africans’ interest in hygiene and sewage. The strength of correlations ranged from weak to strong. The extracted search volumes did not follow a statistical distribution, which would be significantly different from the normal distribution for the following search terms: sanitation, diarrhoea or diarrhea, hygiene and water or drinking water. The remainder of the search term volumes were statistically significantly different from normal distribution at a 5% level of significance (see Table 1 for details). Therefore the use of the Spearman correlation coefficient is justified to assess the temporal trend in the data for the monthly search volumes, which were related to WASH, in South Africa. The Kruskal-Wallis analysis of variance by ranks indicated that there were statistically significant differences in the median South African public’s interest in the individual WASH-related terms at a 5% level of significance (*H*_c_ = 1582; *p*-value < 0.0001 calculated using Past 3.0). Therefore the search term volumes changed between the individual WASH-related terms and with time in the 2004–2022 period. Time affects the South African public’s interest in WASH-related topics to a varying extent, with the minimum percentage of search volume variance it explains 0.51% for greywater/grey water, and the maximum was 93.41% for wash/WASH.

**TABLE 1:** The statistics of the monthly water, sanitation and hygiene-related search terms.

Statistical parameter	Sanitation	Wash or WASH	Diarrhoea or Diarrhea	Hygiene	Water or Drinking water	Sewage	Greywater or Grey water	Pit latrine	Toilet
Average	1766	2035	30 236	4354	22 902	2103	5203	481	11 149
STD	797	1064	14 845	1344	4781	1022	6998	674	4687
Mode	1900	1650	38 700	4000	21 900	1850	0	0	13 150
Median	1775	1650	29 750	4200	22 050	1850	2885	355	12 575
Average COV (%)	22 ± 6	9.2 ± 1.6	21 ± 4	9.0 ± 6.7	5.4 ± 0.8	11 ± 3	18 ± 10	9.0 ± 7.8	1.3 ± 1.3
Spearman correlation coefficient	0.25769	0.96647	0.75115	−0.29462	0.69421	−0.25702	0.07164	0.55473	0.80968
Variance percentage†	6.64	93.41	56.42	8.64	48.19	6.61	0.51	30.92	65.12
*p*	0.0001	0	0	0.00001	0	0.00011	0.28789	0	0
Kolmogorov-Smirnov test statistic	The value of the K-S test statistic (D) is 0.06771	The value of the K-S test statistic (D) is 0.14598	The value of the K-S test statistic (D) is 0.06662	The value of the K-S test statistic (D) is 0.08981	The value of the K-S test statistic (D) is 0.08652.	The value of the K-S test statistic (D) is 0.14693	The value of the K-S test statistic (D) is 0.22807	The value of the K-S test statistic (D) is 0.23719	The value of the K-S test statistic (D) is 0.13786
Kolmogorov-Smirnov *p*	0.24981	0.00014	0.26609	0.05228	0.06783	0.00012	< 0.00001	< 0.00001	0.0004
Distribution	Not statistically significantly different from normal distribution	Statistically significantly different from normal distribution	Not statistically significantly different from normal distribution	Not statistically significantly different from normal distribution	Not statistically significantly different from normal distribution	Statistically significantly different from normal distribution	Statistically significantly different from normal distribution	Statistically significantly different from normal distribution	Statistically significantly different from normal distribution

Note: Extracted from Google using the Keywordseverywhere.com plugin.

COV, coefficient of variation; STD, standard deviation.

† , Percentage of dependent variable variance as explained by the independent variable.

### Links between the search trends in the water, sanitation and hygiene-related terms in South Africa, access to water, sanitation and hygiene resources and the burden of WASH-related diseases

The *YTWRMT* data were calculated based on the raw data from the World Bank data, and the results are shown in Table 2 for the 2004–2020 period. That time period was based on the data availability, and there were some challenges encountered. Access to hand washing facilities was not a standard parameter that either Statistics South Africa or the World Bank collected on a constant basis between 2004 and 2020.

**TABLE 2:** The *YTWRMT* values and the World Bank data for the WASH impacts or landscape in South Africa from 2004 until 2020.

Calendar year	YTWRMT (times)	ATBW† (%)	ATBS‡ (%)	ATHWF§ (%)	MCUFRY (deaths per 1000 live births in South Africa)	RDWDR (Death reduction per unit improvement of the population’s WASH situation)
2004	862 860	86.641	62.975	Not reported; 0¶	77.7	115.4
2005	830 175	87.164	64.016	Not reported; 0¶	78.8	115.8
2006	721 795	87.678	65.048	Not reported; 0¶	79.2	115.2
2007	650 083	88.183	66.072	Not reported; 0¶	75.3	108.5
2008	736 690	88.678	67.086	Not reported; 0¶	68.9	98.3
2009	676 660	89.163	68.091	Not reported; 0¶	60.3	85.2
2010	836 685	89.638	69.087	Not reported; 0¶	52.0	72.8
2011	766 485	90.105	70.072	Not reported; 0¶	45.6	63.3
2012	802 045	90.562	71.048	43.3	41.5	53.9
2013	817 385	91.009	72.014	61.8	39.3	49.4
2014	907 155	91.447	72.969	62.5	37.6	46.9
2015	1 043 335	91.876	73.914	62.6	36.3	44.9
2016	1 151 075	92.296	74.848	63.3	35.2	43.2
2017	1 224 780	92.707	75.771	63.0	34.6	42.1
2018	1 281 265	93.109	76.683	55.8	33.9	41.3
2019	1 250 560	93.501	77.584	55.0	33.0	40.0
2020	1 362 510	93.885	78.475	58.7	32.2	38.6

Note: Extracted from Google using the Keywordseverywhere.com plugin.

WASH, water, sanitation and hygiene; YTWRMT, total search volume of the WASH-related terms; ATBW, percentage of the South African population with access to basic water; ATBS, percentage of the South African population with access to basic sanitation facilities; ATHWF, percentage of the South African population with access to hygiene and hand-washing facilities; MCUFRY, mortality of children under 5 years of age; RDWDR, represents the decrease in the rate of diarrhoeal diseases per unit improvements in the WASH situation of the South African population.

† , Access to basic water as percentage of the South African population.

‡ , Access to basic sanitation as percentage of the South African population.

§ , Access to hand-washing facilities as percentage of the South African population which were averaged values from the local and international sources, or the single available values were used only.

¶ , Data not reported or not collected; a 0 is assigned for the calculation purposes.

No data on hand washing (facility availability), as a semi-proxy measure of hygiene adherence or access by the South African population were available from either the World Bank or from Statistics South Africa from 2004 until 2011. Both sources reported some indicators for the 2012–2020 period, and those were averaged with averages shown in Table 2. The correlation analyses indicated that the Spearman correlation coefficient between *YTWRMT* and the South African population’s access to basic water and basic sanitation was always equal to 0.78431 in both cases. This correlation was statistically significant at a 5% level of significance, as the *p*-value was equal to 0.00019. Therefore access to basic water and basic sanitation explain about 61.5% of the variability in the *YTWRMT* values. The correlation analyses indicated that the Spearman correlation coefficient between *YTWRMT* and the South African population’s access to hand-washing facilities was always equal to –0.01667. That correlation was not statistically significant at a 5% level of significance as the *p*-value was equal to 0.96605.

Therefore the *YTWRMT* values were likely related to the access of the South African population to improved water and sanitation, but they were not related to the hand-washing facilities access. The Spearman correlation coefficient between *YTWRMT* and the *MCUFRY* values was equal to –0.81863. Therefore the *MCUFRY* values as a measure of the health management of waterborne and WASH-related diseases explained 67% of the variability in *YTWRMT*, and the *YTWRMT* values were inversely proportional to *MCUFRY*. This correlation was statistically significant at a 5% level of significance, as the *p*-value was equal to 0.00006. The *MCUFRY* values have been decreasing in South Africa with time between 2004 and 2020. The government has made significant strides in achieving this. Therefore, the correlation results indicate that the increasing interest of the South African public in WASH-related terms correlated with the improvements and decreases in the disaster risk from waterborne diseases in the country. This could indicate that the awareness about the WASH-related topics increases as the disaster risk related to them decreases. Such a trend could be an indication of the increased awareness and receptiveness of the South African public to the WASH-related disaster risk reduction campaigns and general awareness public health campaigns on the subjects related to water, sanitation and hygiene. The comparable percentage of the variance in *YTWRMT* by *MCUFRY* and the access to improved water and sanitation is likely the result of the partial correlation between these variables (Tandlich et al. 2024). This indicated that a more detailed examination of the *YTWRMT* values to the independent variable was necessary, and it is performed in the ‘Wider context and interpretation of the findings on *YTWRMT*’ below.

### Wider context and interpretation of the findings on *YTWRMT*

Access to improved water and sanitation of the South African population was shown to have a limited impact on the *MCUFRY* values in South Africa (Tandlich et al. 2024). This was confirmed in a similar study and in a wider context of sub-Saharan Africa recently (Gaffan et al. 2023:1136299). However, the situation will be slightly different when examining the possible relationship between the *MCUFRY* values in South Africa and the country population’s interest in WASH-related topics. This is based on the fact that access to improved drinking water and sanitation will have a direct impact on the everyday quality of life of South Africans. At the same time, there have been tragic deaths related to dilapidated sanitation infrastructure. For example, news stories of pupils drowning in pit toilets are available in the public domain (Mahopo 2017). These incidents led to the filing of court cases against government officials for negligence after the death of a pupil in a school in the Eastern Cape Province of South Africa (SALFII 2020). In that case, the judge did not find that the government’s failure to upkeep toilets at the school led to the death of a pupil (SALFII 2020). However, it is likely that a member of the South African public would look at the dilapidated toilet as the cause of the child’s death. This, combined with the significance of access to water and sanitation in the daily lives of South Africans, is definitely a justification to expect that a correlation will exist between the *YTWRMT* values and the overall WASH situation faced by the South African population. To reflect this overall situation, and to take any possible covariance or mutual relationships into account between *MCUFRY* and other independent variables from Table 2 account, a composite criterion was defined to holistically reflect the drivers of the South African public interest in the WASH-related topics.

This criterion is defined as the rate of decrease in the WASH-related disaster risk because of the improvement in the access of South Africans to basic or improved water, sanitation and hygiene. The definition of *RDWDR* represents the decrease in the rate of diarrhoeal diseases per unit improvements in the WASH situation of the South African population as shown in Equation (4):


$$RDWRD=100\times \frac{MCUFRY}{0.45\times ATBW+0.45\times ATBS+0.1\times ATHWF}$$ [Eqn 4]

where:

*RDWDR* = represents the decrease in the rate of diarrhoeal diseases per unit improvements in the WASH situation of the South African population.

*MCUFRY* = represents the mortality among children under 5 years of age (data is reported as number of deaths per 1000 live births).

*ATBW* = the percentage of the South African population with access to basic water.

*ATBS* = the percentage of the South African population with access to basic sanitation facilities.

*ATHWF* = the percentage of the South African population with access to hygiene and hand-washing facilities.

The denominator is expressed as a weighted average of the access to water, sanitation and hand washing or hygiene. The weighting factors are based on the estimates of the authors as experts on WASH, and they take into account the correlation analysis results. Therefore access to improved water and sanitation was given a weighting factor of 0.45, while the hand washing facility parameter was given a weighting factor of 0.10. The coefficient 100 in Equation (4) provides for a conversion of the percentages in the denominator to dimensionless numbers. The Spearman correlation coefficient between *YTWRMT* and *RDWDR* was equal to –0.78922, and the correlation was statistically significant at a 5% level of significance (*p*-value = 0.00017). The *RDWDR* value therefore explained 62.3% of the variability in the South African public’s interest in WASH-related terms. Therefore, it is clear that the improving WASH situation in South Africa leads to a higher interest of the country’s population in the subject.

The increase in access to basic water and sanitation facilities is in line with the policy positions of the National Sanitation Policy of 2016 (DWS 2016). In this policy, section 2.1.3. contains the following text: ‘Basic services are a human right and “Some for All”, rather than “All for Some”’ (DWS 2016:section 2.1.3 and page 13). At the same time, the improvements in the WASH situation in the country contributed to the ‘universal access to sanitation in human settlement areas needs to be planned and implemented as part of the holistic human settlement-wide plan’ (DWS 2016:section 2.1.3 and page 13). Improvement in the WASH situation in South Africa is in line with the integrated nature of the planning and service delivery on sanitation in the country (DWS 2016:section 3.1.3 and page 20). Donga et al. (202112552) reported on the survey results from the Western Cape Province of South Africa at the household level during the COVID-19 pandemic. The authors reported that there was an increased focus on the family and the importance of hygiene, awareness about and habits related to it in households (Donga et al. 2021:12552). This could indicate that increased interest and awareness in WASH from the pre-pandemic period could have made the South African public more resilient and able to cope with the novel risks and disaster settings during the COVID-19 pandemic. Such coping ability and awareness could provide a foundation for increased preparedness and mitigation campaigns against other than WASH-related infectious diseases. From the point of viewpoint, it is necessary to understand qualitative drivers in the WASH-related Google searches by the South African public. This is examined below.

### Qualitative drivers of the South African public’s interest in WASH over various time periods

The Keywordseverywhere.com plugin provides additional information about the terms that people who searched, for example hygiene, were also interested in. This can provide an indication of the factors that trigger the search for individual terms by the South African population. One term from the nine WASH-related terms was chosen as a test case for the qualitative drivers. The first extraction was done for hygiene for the 2004–2022 period. The related keywords, as well as other qualitative drivers, are shown in Table 3 to Table 5. As can be seen from Table 3, the related terms to hygiene did not change between the three time periods. Specific topics to be covered in public awareness campaigns could include hygiene products, the relationship between and hygiene, along with the other terms identified in Table 3. The information can be complemented by the information on the qualitative drivers that the ‘people also searched for’ in South Africa in relation to hygiene (see Table 4 for details). Here, it is clear that common topics such as ‘hygiene products’, ‘good hygiene’ and ‘types of hygiene’ are starting to emerge as common qualitative interest drivers. Finally, the long-tail keywords as qualitative drivers indicate that there is an overlap with the previous two driver categories (see Table 5 for details). The dominant and common terms again include ‘hygiene products’, and ‘hygiene types/types of hygiene’, but new terms are added such as the meaning of hygiene. Combining the additional information, that the plugin extracts from the WASH-related Google searches by South Africans, can provide a more complete picture for targeting specific topics in preparedness and mitigation campaigns.

**TABLE 3:** The ‘related keywords’ qualitative drivers that South Africans searched for using the Google engine in relation to hygiene from January 2004 until June 2022.

Keywords number	2004–2022†	2017–2022†	June 2021–June 2022†
1	Hygiene products	Hygiene products	Hygiene products
2	Health and hygiene	Health and hygiene	Health and hygiene
3	Types of hygiene	Types of hygiene	Types of hygiene
4	Good hygiene	Good hygiene	Good hygiene
5	Importance of hygiene	Importance of hygiene	Importance of hygiene
6	What are the seven practices of personal hygiene	What are the seven practices of personal hygiene	What are the seven practices of personal hygiene
7	Domestic hygiene	Domestic hygiene	Domestic hygiene
8	10 importance of hygiene	10 importance of hygiene	10 importance of hygiene

Note: Extracted from Google using the Keywordseverywhere.com plugin.

† , The time periods are presented for which the ‘related keywords’ qualitative drivers of the WASH-related interest among the South African public were extracted.

**TABLE 4:** The ‘people also searched for’ qualitative drivers that South Africans searched for using the Google engine in relation to hygiene from January 2004 until June 2022.

Keywords number	2004–2022†	2017–2022†	June 2021–June 2022†
1	Hygiene products	Hygiene products	Hygiene products
2	Types of hygiene	Types of hygiene	Types of hygiene
3	Good hygiene	Good hygiene	Good hygiene
4	Importance of hygiene	Importance of hygiene	Importance of hygiene
5	Hygiene practices	Hygiene practices	Hygiene practices
6	Hygiene in hindi	Hygiene in hindi	Hygiene in hindi

Note: Extracted from Google using the Keywordseverywhere.com plugin.

† , The time periods are presented for which the ‘people also searched for’ qualitative drivers of the WASH-related interest among the South African public.

**TABLE 5:** The ‘long-tail keywords’ qualitative drivers that South Africans searched for using the Google engine in relation to hygiene from January 2004 until June 2022.

Keywords number	2004–2022†	2017–2022†	June 2021–June 2022†
1	Hygiene meaning	Hygiene meaning	Hygiene meaning
2	Personal hygiene	Personal hygiene	Personal hygiene
3	Sleep hygiene	Sleep hygiene	Sleep hygiene
4	Five moments of hand hygiene	Five moments of hand hygiene	Five moments of hand hygiene
5	Hygiene factors	Hygiene factors	Hygiene factors
6	Hygiene products	Hygiene products	Hygiene products
7	Hand hygiene	Hand hygiene	Hand hygiene
8	Hygiene definition	Hygiene definition	Hygiene definition
9	Hygiene types	Hygiene types	Hygiene types

Note: Extracted from Google using the Keywordseverywhere.com plugin.

† , The time periods are presented for which the ‘long-tailed keywords’ qualitative drivers of the WASH-related interest among the South African public.

How representative the Keywordseverywhere.com plugin data are, or what sample size they represent, is analysed below. Results are linked to preparedness and practical data applications of the desktop and modelling studies on the WASH-related public interest in South Africa, over various times.

### Sample size estimation of the Google search terms

Results of the sample size estimations for the Keywordseverywhere.com plugin from Google are shown in Table 6. All data were used as extracted from the particular data source. At the same time, the values for 2021 and 2022 were extrapolated based on the percentage yearly increase for the 2016–2020 period. The WASH-related searches on Google represented an ever-decreasing percentage of the total estimated Google searches in South Africa between 2004 and 2022. The maximum value was observed in the first year of this time period with a value of 0.245%, and it decreased steadily to 0.008% in 2022. One of the reasons for the low sample sizes could be the low Internet access coverage in South Africa, which only reached 10% by 2009. Until 2009, the internet access coverage was low and independent of time and therefore the data prior to 2009 should be seen as having limited statistical and information power. At the same time, the usage of the Internet reached 70% of the South African population by the onset of the COVID-19 pandemic in 2020 and so some limitations still remain in the data at present day.

**TABLE 6:** The estimation of the sample size of the *YTWRMT* values as the fraction of the estimated total number of Google searches conducted in South Africa from January 2004 until June 2022.

Calendar year	YTWRMT (times)	TGSGY (number of searches)	TWP (number of human beings)	TPSA (number of human beings)	PWPIA (%)	PSAPIA (%)	PTSSA (%)	NSS†
2004	862 860	7.95 × 10^10^	6.43 × 10^9^	47 291 610	14	8.43	0.245	385/0.0008
2005	830 175	1.41 × 10^11^	6.51 × 10^9^	47 880 595	16	7.49	0.171	385/0.0008
2006	721 795	2.31 × 10^11^	6.59 × 10^9^	48 489 464	17	7.61	0.095	385/0.0008
2007	650 083	3.72 × 10^11^	6.67 × 10^9^	49 119 766	20	8.07	0.059	385/0.0008
2008	736 690	6.11 × 10^11^	6.76 × 10^9^	49 779 472	23	8.43	0.045	385/0.0008
2009	676 660	7.04 × 10^11^	6.84 × 10^9^	50 477 013	26	10.00	0.034	385/0.0008
2010	836 685	1.16 × 10^12^	6.92 × 10^9^	51 216 967	29	24.00	0.012	385/0.0008
2011	766 485	1.42 × 10^12^	7.00 × 10^9^	52 003 759	31	33.97	0.007	385/0.0007
2012	802 045	1.21 × 10^12^	7.09 × 10^9^	52 832 659	34	41.00	0.007	385/0.0007
2013	817 385	2.16 × 10^12^	7.18 × 10^9^	53 687 125	36	46.50	0.004	385/0.0007
2014	907 155	2.10 × 10^12^	7.26 × 10^9^	54 544 184	38	49.00	0.004	385/0.0007
2015	1 043 335	2.83 × 10^12^	7.35 × 10^9^	55 386 369	40	51.92	0.004	385/0.0007
2016	1 151 075	2.00 × 10^12^	7.43 × 10^9^	56 207 649	43	54.00	0.006	385/0.0007
2017	1 224 780	2.23 × 10^12^	7.52 × 10^9^	57 009 751	46	56.17	0.006	385/0.0007
2018	1 281 265	2.23 × 10^12^	7.60 × 10^9^	57 792 520	49	62.40	0.006	385/0.0007
2019	1 250 560	2.04 × 10^12^	7.68 × 10^9^	58 558 267	54	68.20	0.006	385/0.0007
2020	1 362 510	2.23 × 10^12^	7.76 × 10^9^	59 308 690	60	70.00	0.007	385/0.0006
2021	1 227 670	2.23 × 10^12^	7.84 × 10^9^	60 114 695	65	74.00	0.006	385/0.0006
2022	1 519 110	2.23 × 10^12^	7.92 × 10^9^	60 931 654	70	79.00	0.008	385/0.0006

Note: Extracted from Google using the Keywordseverywhere.com plugin.

TGSGY, the total number of all Google searches performed in a given calendar year worldwide; TPSA, the total South African population in a given calendar year; TWP, the total global population in a given calendar year; PSAPIA, the percentage of the South African population who had access to the Internet or who were using the Internet in a given calendar year in Equations (2) and (3); PWPIA, the percentage of the world population, which used the Internet in a given calendar year; PTSSA, the percentage of all Google searches in South Africa in a given year; NSS, necessary sample size.

† , The necessary sample size as calculated using the online calculator at https://www.calculator.net/sample-size-calculator.html (website accessed on 24th September 2022). The absolute values of the sample size needed are calculated, as are the percentages of the total population.

The increasing amount of information on the Internet and the diversifying interests of the South African population could provide a partial answer for the data in Table 6. The search volumes extracted from Google using the Keywordseverywhere.com plugin, based on the single search per user assumption, exceed the necessary sample size for a representative survey, as based on the data in the two right-most columns in Table 6. Based on these calculation results, the Keywordseverywhere.com plugin can be used to follow and estimate the public’s interest in WASH-related topics in South Africa in the future. The continued interest of the South African public in WASH-related terms indicates that there is a need for continued research into sewage, drinking water, greywater, hygiene and sanitation in South Africa.

## Conclusion

Results of the current study thus indicate that low-cost Google-linked plugins can be a useful tool in the assessment of the public’s interest in WASH-related topics and its drivers in South Africa. This is supported by the fact that the monthly Google volumes for the WASH-related keywords increased with improving access to improved water, sanitation and hygiene resources. That increase was accompanied by a drop in the disaster risk proxy indicators for waterborne/hygiene-related diseases. The number of searches provides a representative sample size if the Google searches are considered a surrogate for preliminary/limited surveys of the South African public’s interest in WASH-related topics. Qualitative drivers of the South African public’s interest in WASH are also extractable from Google by the Keywordseverywhere.com plugin.

## Appendix 1

**TABLE 1-A1:** The source information for the access to hand washing facilities from the General Household Survey from Statistics South Africa for the 2004–2020 time period.

Calendar year	Page and Table or Figure for data extraction	Access to hand-washing facilities as a percentage of the population	General Household Survey website	Access to hand-washing facilities as a percentage of the population according to the World Bank§
2012	Not applicable	Not reported	https://www.statssa.gov.za/publications/P0318/P03182012.pdf	43.3
2013	Figure 37 on page 48	80.0†	https://www.statssa.gov.za/publications/P0318/P03182013.pdf	43.5
2014	Figure 37 on page 50	81.3†	https://www.statssa.gov.za/publications/P0318/P03182014.pdf	43.6
2015	Figure 49 on page 46	81.5†	https://www.statssa.gov.za/publications/P0318/P03182015.pdf	43.7
2016	Figure 45 on page 45	82.7†	https://www.statssa.gov.za/publications/P0318/P03182016.pdf	43.9
2017	Figure 45 on page 43	82.1†	https://www.statssa.gov.za/publications/P0318/P03182017.pdf	44.0
2018	Figure 11.5 on page 52	67.5‡	https://www.statssa.gov.za/publications/P0318/P03182018.pdf	44.1
2019	Figure 11.4 on page 45	65.9‡	https://www.statssa.gov.za/publications/P0318/P03182019.pdf	44.2
2020	Figure 10.4 on page 38	72.9‡	https://www.statssa.gov.za/publications/P0318/P03182020.pdf	44.4

† , Based on subtraction of the percentage of the population without access to water for washing hands from 100%. In more detail, these were extracted with no water to wash hands. At the same time, the General Household Survey was also searched for hygiene, soap and wash hands.

‡ , The percentage of the South African population that had access to hand washing facilities (with soap).

§ , Data were extracted from the World Bank Data (2022d).
